# Systematic evaluation of subgroup analyses of inhaled treprostinil in pulmonary hypertension due to interstitial lung disease

**DOI:** 10.1371/journal.pone.0318739

**Published:** 2025-02-12

**Authors:** Pablo Martínez-Puig, Nerea Báez-Gutiérrez, Héctor Rodríguez-Ramallo, Laila Abdelkader-Martin, Remedios Otero-Candelera

**Affiliations:** 1 Hospital Pharmacy Department, Reina Sofía University Hospital, Cordoba, Andalusia, Spain; 2 Hospital Pharmacy Department, Virgen Macarena University Hospital, Seville, Andalusia, Spain; 3 Hospital Pharmacy Department, Virgen del Rocio University Hospital, Seville, Andalusia, Spain; 4 Unidad Médico Quirúrgica de Enfermedades Respiratorias, Quirúrgica, Instituto de Biomedicina de Sevilla (IBIS), Hospital Universitario Virgen del Rocío/Universidad de Sevilla, Seville, Spain; Kurume University School of Medicine: Kurume Daigaku Igakubu Daigakuin Igaku Kenkyuka, JAPAN

## Abstract

**Background:**

The INCREASE trial introduced a novel therapeutic option for Pulmonary Hypertension caused by Interstitial Lung Disease. Subsequently to this trial, several subgroup analyses were conducted, aiming to explore specific effects within subgroups.

**Objective:**

This study aimed to evaluate the subgroup analyses performed in the INCREASE trial and to identify potentially reliable subgroup effects.

**Methods:**

A methodological assessment of the subgroup analyses was performed. Claims of subgroup effect were evaluated using three different tools: Sun, X et al. 2012, Gil-Sierra, M.D et al. 2020, and Schandelmaier, S et al. 2020. Additionally, all statistically significant subgroup effects that were not claimed by the authors were evaluated.

**Results:**

Five claims of subgroup effect were identified; none of them achieved statistical significance when assessed using an interaction test. The evaluation conducted with the three tools consistently yielded very low credibility for all the claims. During the assessment, a statistically significant subgroup effect of moderate credibility was identified, which the authors did not claim: iTre appeared to improve exercise capacity exclusively in patients with Pulmonary Vascular Resistance ⋝ 4 WUs.

**Conclusions:**

Due to methodological limitations, the credibility of subgroup claims from the authors of the INCREASE was lacking and, therefore, should not be relied upon to inform decisions on an individual basis.

## Introduction

Pulmonary hypertension (PH) is a rare pathophysiological disorder with diverse etiology or rarely, occurring without an apparent cause [1]. Given the complexities associated with diagnosing and treating PH, a comprehensive clinical classification has been developed to categorize PH based on clinical presentation, findings, underlying conditions, and treatment [2]. Among the five groups included in this classification, PH associated with lung diseases or hypoxia is currently classified under group 3 [1, 3]. Some of the respiratory diseases commonly associated with group 3 PH include chronic obstructive pulmonary disease (COPD), idiopathic interstitial lung diseases (ILD), including connective tissue disease-associated ILD (CTD-ILD), and idiopathic pulmonary fibrosis (IPF) [4, 5]. Due to the mixed etiology of group 3 PH, estimating the prevalence of this disorder is challenging. In patients with COPD, the prevalence ranges from 30 to 70% of patients [6], whereas, in IPF, it varies from 8 to 15% at initial diagnosis and increases to 86% at advanced stages [7, 8].

PH associated with lung diseases or hypoxia represents the second leading cause of PH [9, 10], with reported mortality rates among the highest in any diagnostic group [11, 12]. Despite the poor clinical prognosis and the prevalence of PH associated with lung diseases or hypoxia, treatment options are limited, and adjunctive therapies, including the treatment of the underlying disease and clinical stabilization, remain the standard of care [1]. Clinical trials investigating the pharmacological management of group 3 PH have faced challenges, including conflicting evidence and methodological limitations, such as small sample sizes or short trial durations [13–17]. Moreover, several trials assessing drugs recommended for pulmonary arterial hypertension (PAH) have yielded negative results, ranging from no benefit when compared to placebo in 6-minute walk distance (6MWD) and perceived well-being [18–21] to detrimental results for relevant outcomes such as increased risk of clinical worsening and potential excess mortality [13, 22–26].

One of the frequently targeted pathways in the treatment of PAH is the activation of the prostacyclin metabolic pathway. This pathway induces direct vasodilation, exhibits antiproliferative activity and inhibits platelet aggregation [27–31]. Treprostinil, a prostacyclin analogue, activates the prostacyclin metabolic pathway and directly affects pulmonary circulation when administered by the inhaled or intravenous route. Inhaled treprostinil (iTre) has also been suggested to possess antifibrotic properties, including the reduction of fibrocyte recruitment to sites of vascular remodelling, suppression of profibrotic fibroblast activity, and inhibition of fibronectin and collagen synthesis and deposition [32, 33]. In the context of PH group 1, treatment with iTre has shown to improve exercise capacity and enhance quality of life in patients receiving bosentan or sildenafil [34].

In the case of group 3 PH patients, preliminary results from underpowered studies and observational data have suggested potential benefits of iTre [35–37]. Based on these preliminary results, the INCREASE trial authors reported that iTre improved exercise capacity, assessed as peak 6MWD from the baseline to week 16, in patients with PH due to interstitial lung disease [38]. This result suggests that patients with PH associated with lung diseases or hypoxia may finally have a PH-specific therapy that improves their outcomes.

Data from Randomized Controlled Trials (RCTs), such as the INCREASE trial, play a crucial role in guiding individual treatment decisions for PH. However, the findings of RCTs are reported as average results, and especially in rare diseases such as PH, the patients included are chosen from heterogeneous populations. To inform decisions on an individual level, more detailed information is required. Subgroup analyses are usually performed to assess whether the effect of the intervention will change due to the patient’s baseline characteristics, such as sex, age, or disease severity. Researchers may claim differences in treatment effects between subgroups based on the findings of these analyses. However, subgroup claims interpretation should be carefully worded since misleading claims about subgroup effects could prevent patients from receiving necessary therapies or possibly cause them to receive ineffective or even harmful treatments [39–41]. In order to help with the interpretation of claims of subgroup effects, explicit criteria have been developed to provide readers of medical literature with tools to assess the reliability of subgroup analyses [42–47].

This study aimed to perform a methodological assessment of the subgroup analyses conducted in the INCREASE trial with the following objectives: 1) Evaluate the credibility of the claims of subgroup effects made by the authors of the manuscript. 2) Identify potential subgroup effects that may not have been detected or acknowledged by the manuscript authors and evaluate their credibility.

## Materials and methods

### Literature search

A systematic search was conducted in MEDLINE to identify all publications containing subgroup analyses related to the results of the INCREASE trial.

The search was conducted in September 2022 and included terms such as “increase trial”, “treprostinil”, “pulmonary hypertension”, “interstitial lung disease”, and “chronic obstructive pulmonary disease”. Clinicaltrials.gov was consulted to identify publications related to NCT02630316.

Supplementary material and the trial protocol were included and consulted to verify the prespecified nature, number, and characteristics of subgroup analysis.

### Data extraction and synthesis

The included articles were examined to determine whether subgroup analyses have been conducted. A subgroup effect was defined as a disparity in the magnitude of a treatment effect in a specific group of a study population [48]. A subgroup factor was defined as a study variable by which the population may be classified into distinct subgroups defined by patients’ characteristics. A subgroup analysis was defined as an analysis comparing categories within a subgroup factor. For example, the comparative analysis of the subgroups within the gender factor: male vs female. A subgroup effect claim was defined as an author statement in the manuscript’s abstract or discussion sections, reporting that the intervention effect varied between two or more categories of a subgroup factor. The following data were collected:

Reporting of subgroup analysis: Number of subgroup factors, number of subgroup analyses, number of outcomes for which subgroup analyses were conducted, use of forest plots, prespecified or post hoc analyses, and the statistical method used to assess the heterogeneity of the treatment effect (descriptive, subgroup P-values and confidence interval or a statistical test for interaction). When assessing the credibility of subgroup claims, the number of factors and subgroup analyses were counted additively to those reported in previously published manuscripts.

Claims of subgroup effects: the number of subgroup claims and the subgroup variables (primary or secondary outcome) were recorded. Claims of subgroup effects were classified into the following categories based on Sun et al. classification [43]: strong claim, claim of a likely effect, or suggestion of a possible effect.

Subgroup analyses outcomes were classified as follows:

1. Primary outcome: when the variable was the primary endpoint of the INCREASE trial.
2. Secondary outcomes: endpoints predefined in the protocol and reported in the main results of the INCREASE trial.
3. Exploratory outcomes: endpoint designed at post hoc.

Missing information: When any of the variables collected were not specified in the manuscript, they were classified as ’unclear’. For analysis purposes, when assessing Sun’s criteria and claim’s strength, variables with unclear inputs were considered as follows:

- Negative for dichotomous qualitative variables. The absence of a critical variable to evaluate subgroup analysis was considered inadequate reporting of RCT methods. Unclear inputs were shown in the results section for transparency purposes.
- Excluded from the descriptive analysis for quantitative variables.

### Evaluation of subgroup analyses

A pairwise methodological evaluation was conducted for all claims of subgroup effect. Additionally, subgroup effects with statistically significant interaction tests were evaluated regardless of whether they were claimed by the manuscript authors.

When an interaction P value was not provided, a statistical test for interaction was performed using the Joaquin Primo calculator [49]. A subgroup effect was considered statistically significant when the value obtained from the statistical test for interaction was < 0.05. The following tools were applied to evaluate subgroup analysis:

1. Sun et al. 2012 ten criteria used to assess the credibility of subgroup effect [48]: The ten criteria are formulated as questions and are an update of previously published criteria by Yusuf et al. [50] and Oxman and Guyatt [42]. The first four questions evaluate relevant aspects of the study design, while the following two questions examine the analytical aspect, such as using statistical tests for interaction. The remaining four questions explore the specific context of the subgroup effect that may support its credibility. This tool does not provide a final score to guide evaluators, but the credibility of the subgroup analysis is generally based on the overall criteria fulfilled and the evaluator’s interpretation.
2. Checklist for assessing the applicability of subgroup analysis to clinical decision-making [46]: This tool consists of a checklist organized into two parts. The first part includes three preliminary questions that allow rejecting the validity of the subgroup analyses without further analyses. The second part consists of seven criteria that evaluate three key characteristics: i) statistical association, ii) biological plausibility, and iii) consistency. This tool provides a final assessment with a recommendation on the interpretation of the subgroup analysis credibility. The practical applicability of subgroup analyses is classified as probable, possible, doubtful or null.
3. Instrument to assess the Credibility of Effect Modification Analyses (ICEMAN) [47]. This instrument consists of a set of preliminary considerations, five core questions with four response options, and one optional item for additional consideration. The instrument concludes with an overall credibility assessment rated on a visual analogue scale divided into four areas according to the probability that the subgroup effect may present a true heterogeneity of treatment effect; very low credibility (<25%), low credibility (25%–50%), moderate credibility (51%–75%) and high credibility (>75%). Table 1 provides a comparison of the criteria used by all three tools to evaluate the subgroup analysis.

**Table 1:** Methodological criteria to evaluate the credibility of subgroup analysis.

Criteria		Sun X et al. 2012 [48]	Gil-Sierra MD et al. 2019 [46]	ICEMAN [47]
**Study design**	Subgroup analyses prespecification	**✓**	**✓**	✘
	Subgroup factor is a baseline characteristic	**✓**	✘	✘
	Subgroup variable is a stratification factor	**✓**	✘	✘
	Number of subgroup factors analyzed	**✓**	**✓**	**✓**
**Analysis**	Sample size of subgroups populations	✘	**✓**	✘
	Continuous variable or arbitrary cut points	✘	✘	**✓**
	Interaction p value	**✓**	**✓**	**✓**
	Independence of interaction test	**✓**	✘	✘
**Context**	Direction of subgroup effect was prespecified	**✓**	✘	**✓**
	Biological plausibility effect	**✓**	**✓**	✘
	Consistency across related outcomes	**✓**	✘	✘
	Consistency with previous studies	**✓**	**✓**	**✓**
	Overall result of the study	✘	**✓**	✘
	Additional aspects to consider	✘	✘	**✓**

## Results

The search identified four papers related to the INCREASE trial results: the primary analysis [38] and three post hoc studies [51–53].

### Trial characteristics

The INCREASE [38] trial was a multicenter, randomized, double-blind, placebo-controlled trial that evaluated the efficacy of iTre in group 3 HP patients. A total of 326 patients underwent randomization, with 163 assigned to iTre and 163 to placebo. The primary endpoint was the difference between the two groups in the change in peak 6MWD from baseline to week 16. Secondary relevant endpoints included the change in N-terminal prohormone of brain natriuretic peptide (NT-proBNP) levels and clinical worsening, which was measured by the incidence of hospitalization, mortality, transplant, or 15% reduction on 6MWD.

Three post hoc analyses of the INCREASE trial have been reported: 1) Nathan SD et al. 2021 [51] evaluated the changes in forced vital capacity (FVC) in the entire population of the INCREASE trial and among various subgroups defined by the underlying disease or baseline clinical characteristics. 2) Nathan SD et al. 2022 [52] post hoc analysis evaluated the effect of continued treatment with iTre on the impact of multiple disease progression events, defined as at least a 15% decline in 6MWD, exacerbation of underlying lung disease, cardiopulmonary hospitalization, lung transplantation, at least 10% decline in FVC, or death during the duration of the 16-week study. 3) Nathan SD et al. 2022 [53] post hoc analysis categorized patients according to the number of breaths per session (bps; < 9 and ≥ 9 bps) of active drug or placebo attained at four weeks. They assessed clinical worsening (15% decrease in 6MWD, cardiopulmonary hospitalization, lung transplantation, or death) or clinical improvement (15% increase in the 6MWD with 30% reduction NT-proBNP without any clinical worsening event).

### Subgroup analyses

Subgroup analyses were reported in all four papers. Table 2 presents the characteristics of the reported subgroup analysis.

**Table 2:** Characteristics of subgroup analysis reported in the INCREASE trial-related manuscripts.

Reporting of subgroup analysis	Manuscripts			
	Waxman et al. 2021 [38]	Nathan et al. 2021 [51]	Nathan et al. 2022 [52]	Nathan et al. 2022 [53]
Prespecified/post hoc	Prespecified	Post hoc	Post hoc	Post hoc
Statistical methods	Interaction test	Subgroup P value and CI	Subgroup P value	Subgroup P value
Subgroup outcome	Primary	Secondary (Safety)	Exploratory	Exploratory
Forest plot	Yes	No	No	No
N° subgroup factors reported ^a^	7	6 (13)^b^	1 (14)^b^	1 (15)^b^
N° subgroup analysis reported ^a^	7	6 (13)^b^	1 (14)^b^	1 (15)^b^
N° subgroup outcomes	1	1	1	1

CI: confidence interval, LS: Least Square, SE: Standard Deviation,

^a^ Data are presented as the number of subgroup analyses/factors reported in the manuscript. The accumulated number of subgroup analyses/factors is presented in parentheses, considering those published in previous manuscripts.

^b^ The post hoc nature of the subgroup analysis prevents us from knowing the exact number of analyses conducted. Due to this limitation, only the subgroup analyses/factors reported in the manuscript were counted.

This trial’s protocol and statistical analysis plan (SAP) for this trial are available for consultation at clinicaltrials.gov [54]. Additionally, the protocol is available as supplementary material to the manuscript by Waxman et al. 2021 [38]. The SAP specifies that prespecified subgroup analyses will be conducted for the following seven factors: etiology of ILD, baseline walk categories, ILD disease severity as measured by baseline diffusing capacity of the lungs for carbon monoxide (DLCO), sex, pulmonary vascular resistance (PVR), age group, and study drug dose at Week 16.

The number of subgroup factors and subgroup analyses reported in the Waxman et al. 2021 [38] manuscript aligns with those prespecified in the SAP. The actual number of post hoc subgroup analyses conducted is unknown as they are not documented in the trial protocol. We can only identify the post hoc analyses reported in the published manuscripts.

### Claims of subgroup effect

Five claims of subgroup effect were identified in the four papers examined. Waxman et al. 2021 [38] did not claim any subgroup effect. However, differences in treatment effects between subgroups were claimed in all post hoc analyses. Nathan et al. 2021 [51] highlighted three patient subgroups that may experience significant benefits from iTre treatment. Table 3 provides a summary of the analyzed claims of subgroup effects. All five claims were classified as strong claims. The classification of claims based on to their strength is provided in the supplementary material (S1–S6 Tables).

**Table 3:** Subgroup effects claimed by the authors of the INCREASE trial.

N	Ref.	Author claims of subgroup effect
1	[51]	Patients diagnosed with IPF showed the greatest improvement in forced vital capacity with iTre.
2	[51]	Patients with a PVR of ≧ 5.275 WUs showed a greater improvement in FVC with iTre
3	[51]	Patients with a NT-proBNP concentration of ≧503·85 pg/mL showed a greater improvement in FVC with iTre
4	[52]	iTre significantly reduced the risk of multiple disease progression for most major disease subgroups (IIP and CTD-ILD) but not for those patients with CPFE or IPF.
5	[53]	The subgroup of patients that received higher doses of iTre (≥ 9 bps) had better outcomes in preventing clinical worsening and achieving clinical improvement

CTD-ILD: Connective tissue disease- idiopathic lung disease; CPFE: combined pulmonary fibrosis and emphysema; Ref: article in which the subgroup effect was claimed. IPF: Idiopathic pulmonary fibrosis; IIP: idiopathic interstitial pneumonia; iTre: inhaled treprostinil; FVC: forced vital capacity; NT-proBNP: N-terminal prohormone of brain natriuretic peptide; N: Claim number; PVR: pulmonary vascular resistance; Bps: breaths per session.

### Statistically significant subgroup effects unclaimed by the authors

After reviewing the different subgroup analyses performed in the four included papers, a potential subgroup effect was identified in Waxman et al. 2021 [38]: Only for patients with PVR of 4 WU or greater iTre seem to significantly improved exercise capacity from baseline, assessed with 6MWD. A statistical interaction test for this subgroup showed a significant result (p = 0.0009), which remained statistically significant even when adjusted by multiple testing.

### Credibility of subgroup effects

The results of all three evaluation tools for each potential subgroup effect are provided in the supplementary material (S7–S24 Tables). Table 4 collects the main results from the credibility evaluation of subgroup effects.

**Table 4:** Overall results of the evaluation of subgroup analysis from the INCREASE trial.

Dominions		Tool	Claim 1 [51]	Claim 2 [51]	Claim 3 [51]	Claim 4 [52]	Claim 5 [53]	PSEU 1 [38]
**Study design**	**Subgroup analysis prespecification**	Sun	✘	✘	✘	✘	✘	✓
		Gil-Sierra	Null	Null	Null	Null	Null	Probable
	**Subgroup factor is a baseline characteristic**	Sun	✓	✓	✓	✓	✘	✓
	**Subgroup variable is a stratification factor**	Sun	✘	✘	✘	✘	✘	✘
	**Number of subgroup factors analyzed** ^1^	Sun (≤5)	✘	✘	✘	✘	✘	✘
		Gil-Sierra (<9)	Doubtful	Doubtful	Doubtful	Doubtful	Doubtful	Probable
		ICEMAN	DN	DN	DN	DN	DN	DN
**Analysis**	**Sample size of subgroup population**	Gil-Sierra	Probable	Doubtful	Doubtful	Doubtful	Probable	Doubtful
	**Continuous variable, or arbitrary cut points**	ICEMAN	PN/U	PN/U	PN/U	PN/U	PN/U	PN/U
	**Interaction p value** ^2^	Sun	Unclear	✘ (0.11)	✘ (0.30)	Unclear	✘ (0.99)	✓ (0.0009)
		Gil-Sierra	Null	Null	Null	Null	Null	Probable
		ICEMAN	CLE/U	CVLE	CVLE	CLE/U	CVLE	CUE
	**Independence of interaction test**	Sun	N/A	N/A	N/A	N/A	N/A	N/A
**Context**	**Direction of subgroup effect was prespecified**	Sun	✘	✘	✘	✘	✘	✘
		ICEMAN	DN	DN	DN	DN	DN	PN/U
	**Biological plausibility effect**	Sun	✘	✓	✓	✘	✓	✓
		Gil-Sierra	Doubtful	Possible	Possible	Doubtful	Possible	Probable
	**Consistency across related outcomes**	Sun	N/A	N/A	N/A	N/A	N/A	N/A
	**Consistency with previous studies**	Sun	✘	✘	✘	✘	✘	✘
		Gil-Sierra	Doubtful	Doubtful	Doubtful	Doubtful	Doubtful	Doubtful
		ICEMAN	LS/NS/U	LS/NS/U	LS/NS/U	LS/NS/U	LS/NS/U	LS/NS/U
	**Overall result of the study**	Gil-Sierra	Probable	Probable	Probable	Probable	Probable	Probable
	**Overall assessment of ‘statistical association’ criterion**	Gil-Sierra	Null	Null	Null	Null	Null	Probable
	**Additional aspects to consider**	ICEMAN	Decreased ^3^	Decreased ^3^^,^^4^	Decreased ^3^^,^^4^	Decreased ^3^	Decreased ^3^	N/A
**Overall results of the scale**	**Criteria complied/not complied**	Sun	1/8	2/8	2/8	1/8	1/8	4/9
	**Points and credibility**	Gil-Sierra	Null	Null	Null	Null	Null	Doubtful
	**Credibility percentage**	ICEMAN	Very low	Very Low	Very Low	Very low	Very low	Moderate

Sun: Ten criteria used to assess credibility of subgroup effect [48]. Gil-Sierra: Checklist for assessing the applicability of subgroup analysis to clinical decision-making [46]. ICEMAN: Instrument to assess the Credibility of Effect Modification Analyses [47].

Potential subgroup effect unclaimed: PSEU, N/A: Not applicable, CMNE: Chance may not explain, CVLE: Chance a very likely explanation, CLE: Chance a likely explanation, CLE/U: Chance a likely explanation or unclear, CUE: Chance an unlikely explanation, PN/U: Probably no or unclear, LS/NS/U: Little or no support or unclear, DN: Definitely no.

^1^ The number of factors reported in post-hoc studies is added to those found in previous studies.

^2^ P-value was calculated using a statistical test for interaction based on the data provided in the manuscript.

^3^ Statistically significant subgroup analyses from secondary outcomes are likely to be false positive results.

^4^ A different arbitrary threshold was used for the outcome, which deviated from the prespecified analyses.

## Discussion

There are several challenges associated with conducting clinical trials for treatments targeting PH associated with lung diseases or hypoxia. Historically, these trials have yielded a mixture of negative and detrimental results [13, 18–26]. Therefore, investigators of the INCREASE trial deserve commendation for their contributions to the field of PH. Their work has provided valuable insight into this disorder and reported the efficacy of a drug for treating group 3 PH patients.

To address additional research questions regarding the treatment with iTre, the investigators of the INCREASE trial conducted subgroup analyses that were published in several manuscripts. These analyses aimed to find heterogeneity of treatment effects, also denominated subgroup effects, across various subgroup factors such as age, etiology of IDL, or PVR at baseline. Subgroup analyses have the potential to generate investigative hypotheses, pinpoint baseline characteristics that may influence the intervention efficacy or toxicity and assist in tailoring clinical decisions for specific patients [40, 55]. However, addressing the methodological limitations of subgroup analysis is crucial for understanding the implications for personalized care. Subgroup analysis methodological limitations, such as small sample sizes or insufficient control for confounding factors, can oversimplify patient response variability [48]. Such misinterpretations can lead to clinicians avoiding effective treatments over claims of toxicity or administering ineffective treatments based on exaggerated efficacy claims in certain patient subgroups [39–41]. Thus, interpreting these analyses requires caution, especially when applying findings to personalized care.

Detecting significant subgroup effects with robust methodology is infrequent but can be found throughout the literature. Two notable examples of subgroup effects that have had an impact on clinical practice are from the recent Covid19 pandemic. The recovery trial found that dexamethasone resulted in lower 28-day mortality among patients receiving either invasive mechanical ventilation or oxygen alone but not in those not requiring respiratory support [56]. Similarly, the ATTACC, ACTIV-4a, REMAP-CAP and HEP-COVID investigators found that only non-critically ill Covid19 patients benefited from therapeutic dose anticoagulation with low-molecular-weight heparins [57–59]. These findings have significantly influenced treatment approaches for Covid-19 patients based on their specific respiratory support needs and illness severity.

Nevertheless, subgroup analyses can be misleading and produce false results and misguided conclusions [60–63]. A systematic review of subgroup analyses in pulmonary hypertension-specific therapy trials carried out between 2000 and 2020 revealed methodological limitations that undermined their credibility and hindered the advancement of personalized care [64]. The subgroup analyses conducted in the INCREASE trial share similar limitations to those previously reported. Many of these analyses were performed post hoc, utilizing secondary or exploratory outcomes, and relied on statistical analyses without demonstrating a significant interaction test result, which carries a high risk of false positive findings [65]. These methodological choices diminish the certainty of the findings and the claimed differences between subgroups. Paradoxically, the only subgroup analysis with a positive interaction test result, which could have warranted some credibility, was not acknowledged by the authors.

Establishing a priori hypotheses is a crucial step for the planning of statistical analysis and the interpretation of results within a predefined framework, thereby enhancing the credibility and reliability of the findings. Without these hypotheses, analyses become exploratory and are more likely to yield incorrect or false positive results. This problem is exacerbated when multiple hypotheses are tested, elevating the probability of type I errors [40, 65]. The susceptibility of both prespecified and post hoc subgroup analyses to this issue is acknowledged, with post hoc analyses being particularly problematic due to unclear selection criteria and the indeterminate number of subgroups analyzed, which increases the risk of unfounded conclusions [46–48]. Additionally, the likelihood of presenting misleading outcomes is increased by the temptation to perform or selectively report post hoc subgroup analyses influenced by convenient results [50].

While it is true that post hoc subgroup analyses can yield spurious results, they can still be valuable for generating hypotheses if conducted using a rigorous methodology. However, to avoid misleading clinicians and readers, post hoc subgroup analyses should be framed as exploratory or hypothesis-generating analyses and claims of subgroup effects should be carefully written to avoid a false sense of security of the findings. This applies to all subgroup analyses but is particularly relevant for post hoc analyses or analyses based on secondary outcomes.

There is currently no consensus on how the credibility of subgroup analyses should be measured. A systematic survey conducted in 2019 identified 36 unique criteria to evaluate the credibility of such analyses [66]. While experts generally agree on the importance of criteria such as statistical tests for interaction, a priori hypotheses, or biological rationale, there is often disagreement among authors when it comes to evaluating subgroup effects [66]. Additionally, there are no published studies comparing the heterogeneity of credibility assessments obtained from different evaluation tools. In the present study, three tools to assess subgroup analyses’ credibility were applied [46–48], and consistently, the claims of subgroup effects were found to have poor credibility. Although heterogeneity was across the criteria utilized to assess subgroup analyses, the final evaluation for all the subgroup effect statements remained consistent.

Among the potential subgroup effects detected in the INCREASE trial, a possible subgroup effect not claimed by authors was identified in Waxman et al. 2021 [38]. Briefly, only patients with PVR of ≧4 WU seem to benefit from iTre. Among all the hypotheses tested in the included manuscripts, this subgroup effect was the only one that presented moderate credibility. Recent studies suggested that PVR >5 WU is a better threshold for predicting a poorer prognosis in patients with PH associated with ILD and COPD [67, 68]. Based on this finding, current PH guidelines propose PVR to classify patients into non-severe PH (PVR≤5 WU) and severe PH (PVR >5 WU) [1]. It has been shown that patients with severe PH due to lung disease experience worse clinical outcomes compared to those with non-severe PH, indicating the clinical significance of this difference [67–71]. Considering the moderate credibility of this subgroup effect, this finding should be viewed as exploratory and hypothesis-generating, warranting cautious interpretation and further investigation before any clinical application.

Subgroup analyses conducted in clinical trials targeting patients with rare diseases, such as PH, need reliable methodology due to the intrinsic limitations of working with such populations. Rare diseases face several difficulties in drug development, such as small patient populations, limited disease information, and difficulties associated with defining endpoints and biomarkers [72]. The small patient population often corresponds to a limited market size, which may discourage pharmaceutical stakeholders from investing in drug development unless there is a high probability of success, anticipated long-term therapy, or a reasonable pricing strategy [73]. Furthermore, it is unlikely that confirmatory trials will be carried out, and it is improbable to carry out clinical trials to confirm a subgroup effect; therefore, making it more challenging for clinicians to individualize therapy [74]. These challenges may lead healthcare professionals to make treatment decisions based on flawed subgroup analysis results in rare diseases.

In healthcare policy and decision-making, particularly for treatments with significant economic burden like PH therapy, subgroup analyses can prove critical. Such analyses can guide stakeholders to prioritize treatments for subgroups of patients that may receive increased benefits or to avoid treatments for patients with a higher risk of adverse events or those less likely to benefit. However, allocating resources to targeted therapies, which often come with substantial costs, requires evidence from rigorously applied methodologies. Decisions based on subgroup analyses lacking this rigor could lead to inefficient resource use and support for interventions that are not cost-effective. Furthermore, confirmatory studies that seek to confirm results from subgroup analyses are essential to validate its findings for enhancing patient care and the economic efficiency of healthcare interventions [45].

An opportunity to validate some of the results of the subgroup analysis of the INCREASE trial are the currently ongoing TETON trials. These trials consist of two replicated, randomized, placebo-controlled studies, TETON 1 (NCT04708782) and 2 (NCT05255991), which will evaluate the safety and efficacy of inhaled treprostinil for the treatment of IPF for 52 weeks. Currently, only the study design and rationale of the TETON trial are accessible [75], and those not explicitly specify subgroup analyses. However, the complete protocol version, which may contain information regarding subgroup analyses, is not available for review at this time.

### Improvement measures for the future of subgroup analyses

As demonstrated by this analysis, there is substantial room for improvement in subgroup analyses across all three dominions proposed by Sun et al. [48]; design, analysis and context. Additionally, the reporting of subgroup analysis results also requires enhancement. Firstly, critical factors should be considered to improve the credibility of the subgroup analysis results. We emphasize the importance of prespecifying the analyses, the employment of interaction tests, and limiting the number of hypotheses tested for which a biological rationale and the expected direction of effect should always be provided a priori. Implementing these methodological standards would serve as a solid basis to limit the number of false positive results and the misinterpretation of subgroup analysis.

In rare diseases, such as PH-ILD, the small number of available subjects limits statistical power, increasing the risk of unreliable subgroup effects. Therefore, applying these methodological improvements is especially crucial in rare disease trials, where rigorous standards in design, analysis, and reporting are needed to enhance the quality of subgroup findings.

Secondly, authors should be cautious when claiming subgroup effects, as strong claims may create a false sense of confidence in findings that may lack statistical robustness. Enhanced reporting practices and careful interpretation of subgroup effects can provide a more reliable foundation for hypothesis generation and future research.

### Strengths

To the best of our knowledge, this is the first evaluation of the credibility of subgroup analyses carried out in the INCREASE trial. We conducted a comprehensive systematic evaluation of subgroup claims using three distinct tools. While there was considerable heterogeneity in the factors evaluated by the tools, all three were based on standardized criteria specifically designed for this purpose. Moreover, the inclusion of all published studies derived from the trial results enhances the credibility of the analysis presented in this study.

### Limitations

This study presents several limitations. Firstly, the subjective nature of interpreting the scales used to assess claims strength, particularly those without a numerical score, could potentially affect the reliability of our results. While the pairwise work and agreement between researchers improve confidence in our findings, this issue persists because not all potential biases can be mitigated through collaborative evaluation.

Secondly, clinical trials are designed to identify differences in primary outcomes, taking into account risks for type I and II errors, which occur from incorrectly accepting or rejecting the null hypothesis, centered on the primary endpoint [76]. Consequently, a study’s power—its ability to correctly identify an effect—is based on its primary variable. Subgroup analyses for secondary endpoints should be approached with caution due to their higher risk of false positives, necessitating skeptical interpretation to avoid erroneous conclusions. Similarly, tools for evaluating subgroup effects in clinical trials are usually tailored for primary endpoints, and their results should be taken with caution when identifying potentially reliable subgroup effects.

Thirdly, obtaining certain critical variables used in evaluating the credibility of subgroup effects posed challenges, especially for post hoc subgroup analyses. Difficulties often arose from limited information on the specific methodologies employed in addition to difficulties in determining the number of analyses conducted. As unpublished post hoc analyses may exist, this may further undermine the credibility of the subgroup effects identified. Greater transparency in reporting subgroup analyses could improve the reliability of future assessments.

## Conclusions

Although the INCREASE trial provided relevant insights into the efficacy and safety of iTre, the author’s claims of subgroup effect based on the INCREASE trial results lacked credibility and, therefore, should not be used to inform decisions on an individual basis. On the other hand, this study identified a statistically significant subgroup effect of moderate credibility across patients with PVR < 4 WUs who seemed to not benefit from the treatment with iTre. However, this finding should be considered hypothesis-generating rather than a clinical recommendation, warranting cautious interpretation and further investigation in adequately powered studies. Future research in PH should address the methodological limitations highlighted in this analysis to enable robust, clinically meaningful subgroup findings, particularly in rare diseases like PH-ILD.

## Supporting information

S1 Table Criteria for judging the strength of a subgroup claim. (ODT)

S2 Table Strength of subgroup claim 1. (DOCX)

S3 Table Strength of subgroup claim 2. (DOCX)

S4 Table Strength of subgroup claim 3. (DOCX)

S5 Table Strength of subgroup claim 4. (DOCX)

S6 Table Strength of subgroup claim 5. (DOCX)

S7 Table Claim 1 evaluation with Sun et al. 2012 ten criteria used to assess credibility of subgroup effect. (DOCX)

S8 Table Claim 1 evaluation with the Checklist for assessing the applicability of subgroup analysis to clinical decision-making. (DOCX)

S9 Table Claim 1 evaluation with Instrument to assess the Credibility of Effect Modification Analyses. (DOCX)

S10 Table Claim 2 evaluation with Sun et al. 2012 ten criteria used to assess credibility of subgroup effect. (DOCX)

S11 Table Claim 2 evaluation with the Checklist for assessing the applicability of subgroup analysis to clinical decision-making. (DOCX)

S12 Table Claim 2 evaluation with Instrument to assess the Credibility of Effect Modification Analyses. (DOCX)

S13 Table Claim 3 evaluation with Sun et al. 2012 ten criteria used to assess credibility of subgroup effect. (DOCX)

S14 Table Claim 3 evaluation with the Checklist for assessing the applicability of subgroup analysis to clinical decision-making. (DOCX)

S15 Table Claim 3 evaluation with Instrument to assess the Credibility of Effect Modification Analyses. (DOCX)

S16 Table Claim 4 evaluation with Sun et al. 2012 ten criteria used to assess credibility of subgroup effect. (DOCX)

S17 Table Claim 4 evaluation with the Checklist for assessing the applicability of subgroup analysis to clinical decision-making. (DOCX)

S18 Table Claim 4 evaluation with Instrument to assess the Credibility of Effect Modification Analyses. (DOCX)

S19 Table Claim 5 evaluation with Sun et al. 2012 ten criteria used to assess credibility of subgroup effect. (DOCX)

S20 Table Claim 5 evaluation with the Checklist for assessing the applicability of subgroup analysis to clinical decision-making. (DOCX)

S21 Table Claim 5 evaluation with Instrument to assess the Credibility of Effect Modification Analyses. (DOCX)

S22 Table Subgroup effect not claimed evaluated with Sun et al. 2012 ten criteria used to assess the credibility of subgroup effect. (DOCX)

S23 Table Subgroup effect not claimed evaluated with the Checklist for assessing the applicability of subgroup analysis to clinical decision-making. (DOCX)

S24 Table Subgroup effect not claimed evaluated with the Instrument to assess the Credibility of Effect Modification Analyses. (DOCX)
