# Supplementary material for: Systematic evaluation of subgroup analyses of inhaled treprostinil in pulmonary hypertension due to interstitial lung disease
Source: PLoS One. 2025 Feb 12;20(2):e0318739. doi: 10.1371/journal.pone.0318739 (PMC11819518; doi:10.1371/journal.pone.0318739)
Supplement: S4 Table — (DOCX) [file pone.0318739.s004.docx]

**Strength of subgroup Claims.**

**Claim 3**: Patients with a NT-proBNP concentration of ≧503·85 pg/mL showed a greater improvement in FVC with iTre [1]

**Table S4: Strength of subgroup claim 3.**

| **Criteria** | **Reasonably strong claim of a definitive effect** | **Claim of a likely effect** | **Suggestion of a possible effect** |
| --- | --- | --- | --- |
| 1. Did the investigators claim the effect in the abstract? |  |  | ✓ |
| 2. Did the investigators claim the effect in the conclusion of the abstract? |  | ✓ | ✓ |
| 3. Did the investigators claim the effect in the discussion? | ✓ |  | ✓ |
| 4. Did the investigators use descriptive words (e.g. appear/seem to be, may, and might) to soften their statements of the claims? | ✓ |  |  |
| 5. Did the investigators use descriptive words (e.g. particular and special) to strengthen the statement of the claims? |  | ✓ | ✓ |
| 6. Were the authors obviously cautious about the apparent subgroup effect? (e.g. they stated the subgroup effect did not meet some of the important criteria to believe a subgroup effect) | ✓ |  |  |
| 7. Did the investigators indicate the apparent effects need to be explored in future studies (i.e. hypothesis generating)? | ✓ |  |  |
| Overall strength | Strong claim | | |

1. Nathan SD, Waxman A, Rajagopal S, Case A, Johri S, DuBrock H, et al. Inhaled treprostinil and forced vital capacity in patients with interstitial lung disease and associated pulmonary hypertension: a post-hoc analysis of the INCREASE study. Lancet Respir Med. 2021 Nov;9(11):1266–74.
