# Supplementary material for: Systematic evaluation of subgroup analyses of inhaled treprostinil in pulmonary hypertension due to interstitial lung disease
Source: PLoS One. 2025 Feb 12;20(2):e0318739. doi: 10.1371/journal.pone.0318739 (PMC11819518; doi:10.1371/journal.pone.0318739)
Supplement: S5 Table — (DOCX) [file pone.0318739.s005.docx]

**Strength of subgroup Claims.**

**Claim 4:** iTre significantly reduced the risk of multiple disease progression for most major disease subgroups (IIP and CTD-ILD) but not for those patients with CPFE or IPF [1]

**Table S5: Strength of subgroup claim 4.**

| **Criteria** | **Reasonably strong claim of a definitive effect** | **Claim of a likely effect** | **Suggestion of a possible effect** |
| --- | --- | --- | --- |
| 1. Did the investigators claim the effect in the abstract? |  |  | ✓ |
| 2. Did the investigators claim the effect in the conclusion of the abstract? |  | ✓ | ✓ |
| 3. Did the investigators claim the effect in the discussion? | ✓ |  | ✓ |
| 4. Did the investigators use descriptive words (e.g. appear/seem to be, may, and might) to soften their statements of the claims? | ✓ |  |  |
| 5. Did the investigators use descriptive words (e.g. particular and special) to strengthen the statement of the claims? |  | ✓ | ✓ |
| 6. Were the authors obviously cautious about the apparent subgroup effect? (e.g. they stated the subgroup effect did not meet some of the important criteria to believe a subgroup effect) | ✓ |  |  |
| 7. Did the investigators indicate the apparent effects need to be explored in future studies (i.e. hypothesis generating)? | ✓ |  |  |
| Overall strength | Strong claim | | |

1. Nathan SD, Tapson VF, Elwing J, Rischard F, Mehta J, Shapiro S, et al. Efficacy of Inhaled Treprostinil on Multiple Disease Progression Events in Patients with Pulmonary Hypertension due to Parenchymal Lung Disease in the INCREASE Trial. Am J Respir Crit Care Med. 2022 Jan 15;205(2):198–207
