# Supplementary material for: Systematic evaluation of subgroup analyses of inhaled treprostinil in pulmonary hypertension due to interstitial lung disease
Source: PLoS One. 2025 Feb 12;20(2):e0318739. doi: 10.1371/journal.pone.0318739 (PMC11819518; doi:10.1371/journal.pone.0318739)
Supplement: S15 Table — (DOCX) [file pone.0318739.s015.docx]

**Table S15: Claim 3 evaluation with Instrument to assess the Credibility of Effect Modification Analyses.**

| **Schandelmaier S et al. 2020** | **Answer** | **Credibility** |
| --- | --- | --- |
| 1: Was the direction of the effect modification correctly hypothesized a priori? | Clearly post hoc | Definitely no |
| 2: Was the effect modification supported by prior evidence? | No prior evidence | Little or no support or unclear |
| 3: Does a test for interaction suggest that chance is an unlikely explanation of the apparent effect modification? | Interaction p-value >0.05 | Chance a very likely explanation |
| 4: Did the authors test only a small number of effect modifiers or consider the number in their statistical analysis? | Explicitly exploratory analysis or large number of effect modifiers tested | Definitively no |
| 5: If the effect modifier is a continuous variable, were arbitrary cut points avoided? [ ] not applicable: not continuous | Analysis based on cut point(s) of unclear origin | Probably no or unclear |
| 6 Optional: Are there any additional considerations that may increase or decrease credibility? | Subgroup effects from secondary outcomes are likely due to false positive results | Yes, probably decreased |
| 7: How would you rate the overall credibility of the proposed effect modification? | Very low credibility | |
