# Supplementary material for: Systematic evaluation of subgroup analyses of inhaled treprostinil in pulmonary hypertension due to interstitial lung disease
Source: PLoS One. 2025 Feb 12;20(2):e0318739. doi: 10.1371/journal.pone.0318739 (PMC11819518; doi:10.1371/journal.pone.0318739)
Supplement: S16 Table — (DOCX) [file pone.0318739.s016.docx]

**Table S16: Claim 4 evaluation with Sun et al. 2012 ten criteria used to assess credibility of subgroup effect.**

| **Sun et al. 2012 10 criteria** | **Answer** |
| --- | --- |
| **Study design** | |
| 1. Was the subgroup variable a baseline characteristic? | ✓ |
| 2. Was the subgroup variable a stratification factor at randomisation?* | ✘ |
| 3. Was the subgroup hypothesis specified a priori? | ✘ |
| 4. Was the subgroup analysis one of a small number of subgroup hypotheses tested (≤5)? | ✘ |
| **Analysis** | |
| 5. Was the test of interaction significant (interaction P<0.05)? | Unclear |
| 6. Was the significant interaction effect independent if there were multiple significant interactions? | N/A |
| **Context** | |
| 7. Was the direction of subgroup effect correctly prespecified? | ✘ |
| 8. Was the subgroup effect consistent with evidence from previous related studies? | ✘ |
| 9. Was the subgroup effect consistent across related outcomes? | N/A |
| 10. Was there any indirect evidence to support the apparent subgroup effect—for example, biological rationale, laboratory tests, animal studies? | ✘ |
