# Supplementary material for: Systematic evaluation of subgroup analyses of inhaled treprostinil in pulmonary hypertension due to interstitial lung disease
Source: PLoS One. 2025 Feb 12;20(2):e0318739. doi: 10.1371/journal.pone.0318739 (PMC11819518; doi:10.1371/journal.pone.0318739)
Supplement: S23 Table — (DOCX) [file pone.0318739.s023.docx]

**Table S23: Subgroup effect not claimed evaluated with the Checklist for assessing the applicability of subgroup analysis to clinical decision-making.**

| **Gil-Sierra MD et al. 2020** | **Question** | **Response** | **Analysis reliability** | |
| --- | --- | --- | --- | --- |
| I-Statistical association | **Essential questions** |  | |  |
|  | 1. Interaction. Is there interaction, a statistically significant difference between results of different subgroups? | Yes, with P < .05. | | Probable |
|  | 2. Prespecification: Was it prespecified in the method to study that factor? | Yes | | Probable |
|  | **Additional questions** |  | |  |
|  | 3. Subgroup population (N) | N<50 in some arm | | Doubtful |
|  | 4. Number of factors studied: counting prespecified /stratification factors (*) analysed by subgroups in the study | 1-9 prespecified factors analysed | | Probable |
|  | 5. Overall result of study | Positive, but there could be differences among subgroups | | Probable |
|  | Overall assessment of ‘statistical association’ criterion | Possible | | |
| II- Biological plausibility | 6. Is there a plausible hypothesis to explain cause-effect relationship? | It is possible to state a reasonable hypothesis but without a base in previous literature | | Possible |
| III- Consistency | 7. Is there consistency with results obtained in other similar studies? | There are no other studies with which to compare; at least, there is no lack of internal consistency | | Doubtful |

| **Gil-Sierra MD et al. 2020** | **Assessment** | **Score** |
| --- | --- | --- |
| Statistical association | Possible | 2 |
| Biological plausibility | Possible | 2 |
| Consistency | Doubtful | 0 |
| Practical applicability (sum) | Possible | 4 |
