# Supplementary material for: Systematic evaluation of subgroup analyses of inhaled treprostinil in pulmonary hypertension due to interstitial lung disease
Source: PLoS One. 2025 Feb 12;20(2):e0318739. doi: 10.1371/journal.pone.0318739 (PMC11819518; doi:10.1371/journal.pone.0318739)
Supplement: S24 Table — (DOCX) [file pone.0318739.s024.docx]

**Table S24: Subgroup effect not claimed evaluated with the Instrument to assess the Credibility of Effect Modification Analyses.**

| **Schandelmaier S et al. 2020** | **Answer** | **Credibility** |
| --- | --- | --- |
| 1: Was the direction of the effect modification correctly hypothesized a priori? | Vague hypothesis or hypothesized direction unclear | Probably no or unclear |
| 2: Was the effect modification supported by prior evidence? | No prior evidence | Little or no support or unclear |
| 3: Does a test for interaction suggest that chance is an unlikely explanation of the apparent effect modification? | Interaction p-value ≤0.005 | Chance an unlikely explanation |
| 4: Did the authors test only a small number of effect modifiers or consider the number in their statistical analysis? | 4-10 effect modifiers tested | Probably no or unclear |
| 5: If the effect modifier is a continuous variable, were arbitrary cut points avoided? [ ] not applicable: not continuous | Analysis based on cut point(s) of unclear origin | Probably no or unclear |
| 6 Optional: Are there any additional considerations that may increase or decrease credibility? | N/A |  |
| 7: How would you rate the overall credibility of the proposed effect modification? | Moderate | |
