# Supplementary material for: Systematic evaluation of subgroup analyses of inhaled treprostinil in pulmonary hypertension due to interstitial lung disease
Source: PLoS One. 2025 Feb 12;20(2):e0318739. doi: 10.1371/journal.pone.0318739 (PMC11819518; doi:10.1371/journal.pone.0318739)
Supplement: Supplementary file 25 [file pone.0318739.s001.odt]

Criteria for judging the strength of a subgroup claim.

Sun et al. criteria [1] were used to guide reviewers in establishing claims of subgroup effect strength. These criteria are formulated as seven questions about how authors claimed the heterogeneity of effect in the manuscript. According to these prespecified criteria, claims of subgroup effect were categorized as 

- Strong claims of effect: the manuscript authors convey a determined conviction that their findings represent true heterogeneity of treatment effect.

- Claims of a likely effect: the manuscript authors believed that a subgroup effect exists while acknowledging some limitations of their findings.

- Suggestions of a possible effect: the manuscript authors suggested the existence of a subgroup effect but conveyed uncertainty and highlighted the limitations of their findings.

The strength of the claim was established by adding the number of criteria assigned to each category, and finally, the category that fulfils the most criteria was selected. i.e., If the subgroup claim had four out of seven criteria classified as a strong claim, this subgroup claim was classified as a reasonably strong claim of a definitive effect.

Table S1: Criteria for judging the strength of a subgroup claim. 

| Criteria | Reasonably strong claim of a definitive effect | Claim of a likely effect | Suggestion of a possible effect |
| --- | --- | --- | --- |
| 1. Did the investigators claim the effect in the abstract? | Yes | Possible | No |
| 2. Did the investigators claim the effect in the conclusion of the abstract? | Possible* | No | No |
| 3. Did the investigators claim the effect in the discussion? | Yes | Possible | Yes |
| 4. Did the investigators use descriptive words (e.g. appear/seem to be, may, and might) to soften their statements of the claims? | No | Possible | Possible |
| 5. Did the investigators use descriptive words (e.g. particular and special) to strengthen the statement of the claims? | Possible | No | No |
| 6. Were the authors obviously cautious about the apparent subgroup effect? (e.g. they stated the subgroup effect did not meet some of the important criteria to believe a subgroup effect) | No | Some caution possible | Yes |
| 7. Did the investigators indicate the apparent effects need to be explored in future studies (i.e. hypothesis generating)? | No | Possible say desirable to confirm | Yes |

*If a claim appears in the conclusion section of the abstract, it is considered a strong claim. 

- Sun X, Briel M, Busse JW, Akl EA, You JJ, Mejza F, et al. Subgroup Analysis of Trials Is Rarely Easy (SATIRE): a study protocol for a systematic review to characterize the analysis, reporting, and claim of subgroup effects in randomized trials. Trials. 2009 Nov 9;10:101.
